# Evaluation of laser acupuncture versus physical therapy intervention in management of bruxism in children: a randomized controlled trial

**DOI:** 10.1186/s12903-025-05626-x

**Published:** 2025-03-05

**Authors:** Mohamed Farouk Rashed, Myasser Ayman Mohamed, Negm Eldin Ragab Mohamed, Maryam El Mansy

**Affiliations:** 1 Orthodontics and Pediatric Dentistry Department, Oral and Dental Research Institute, National Research Centre, 33 El Buhouth St, Dokki, Giza Governorate, 12622 Egypt; 2 Physical Therapy for Neuromuscular Diseases and Its Surgery, College of Physical Therapy, Misr University for Science and Technology, 26th of July Corridor, First 6th of October, Giza Governorate, 3236101 Egypt; 3 Electrodiagnosis, College of Physical Therapy, Misr University for Science and Technology, 26th of July Corridor, First 6th of October, Giza Governorate, 3236101 Egypt

**Keywords:** Bruxism, Children, Laser, Electromyography, Physical therapy, Sleep hygiene

## Abstract

**Background:**

Bruxism is a predominant behavior in children and is involved in the development of temporomandibular joint (TMJ) disease and myofacial pain. Bruxism can be classified into; sleep and awake bruxism or primary and secondary bruxism. This habit is characterized by a decrease in mouth opening, pain and increased activity of muscles of mastication and loud sounds during sleep. Management includes sleep hygiene, low-level lasers and physical therapy. The aim of this study was to evaluate changes in pain, mouth opening and muscle activity in children with bruxism after using laser acupuncture versus relaxation physical therapy.

**Methods:**

Twenty-four children (6–12 years) with a history of bruxism were randomly allocated to 3 groups of 8 individuals each: Group 1: laser acupuncture; Group 2: physical therapy and Group 3: control. At baseline and after 2 months, the visual analog scale (VAS) score for pain (TMJ) score, maximum degree of mouth opening, and maximum voluntary contraction (MVC) score were recorded.

**Results:**

Compared with the control group, the laser and physical therapy groups presented significant differences in pain, maximum number of mouth openings and MVC (p value are 0, 0.005 and 0 respectively).

**Conclusions:**

Laser acupuncture and physical therapy are promising options for treating SB in children in terms of pain, mouth opening and muscle activity in comparison to sleep hygiene.

**Trial registration:**

The study was registered on ClinicalTrials.gov on November 12, 2023, with ID: NCT06131879.

## Background

Bruxism is one of the tempromandibular joint disorders (TMDs) which are considered as a class of musculoskeletal sicknesses that prejudice the structure and function of this joint [1, 2].

The etiology of bruxism is multidimensional, it may be due to occlusion-related factors (such as occlusal interference and malocclusion), psychological factors (such as stress, anxiety, and socioeconomic issues), and central nervous system originating factors (such as using certain medications like levodopa and antidepressant drugs) [3, 4].

Bruxism can be classified into two chief categories, based on the period of the hyperactivity of muscles of mastication either during sleep; sleep bruxism (SB), or during daytime; awake bruxism [5, 6].

Another classification of bruxism is primary (in which there is no particular medicinal reason) or secondary (which is accompanied by medications intake or certain disorders such as Parkinson’s disease or apnea) [7].

The global prevalence of sleep bruxism is 21%, with the highest occurrence in North America (36% in adults and 28% in children) and the lowest one in Asia (23% in adults and 14% in children). In adult women, the frequency of sleep bruxism was 15%, while in children it was 9%. This suggests that age is a significant factor influencing the prevalence of sleep bruxism among females, on the contrary in males; age does not play a major role in differentiating the prevalence of sleep bruxism among males (8% in adults and 9% in children) [8].

Sleeping bruxism in children is characterized by supplementary symptoms in the daytime, such as headache, earache, pain in the muscles of mastication, a decrease in mouth opening and a loud sound during sleep [9].

Bruxism in children can have several hazards if left unmanaged. These hazards include; enamel wear, leading to tooth sensitivity, microcracks or fractures in teeth and excessive pressure on TMJ and muscle spasm inducing orofacial pain [1].

Nowadays there are several ways to manage SB, such as behavioral strategies and sleep hygiene have been studied. These strategies are non-invasive that can be done at home without need for professional visits. Additionally, occlusal splint therapy (OST) has been used for years for adults and children; however, both of these modalities have restrictions, as their effects for managing bruxism are dependent on the patient’s cooperation and intolerability of children to wear such bulky appliance at young age [10].

Another modality of SB treatment that has been used widely in adults is using medications such as muscle relaxants as a simple and patient-friendly approach. But its use is limited in children because of its proven side effects such as drowsiness, hypotonia, fatigue or weakness and/ or gastrointestinal symptoms [4].

Thus, alternative treatments, such as low-level laser therapy (LLLT), have been developed to replace OST for muscle disorders and have yielded good results. LLLT has been administered to acupuncture points in individuals with temporomandibular disorder, with substantial differences in the signs and symptoms related to the disorder; this technique has good results with respect to muscle performance and strength [11].

Photobiomodulation (PBM) or phototherapy refers to the use of low-intensity light, as light-emitting diodes (LEDs) or light amplification by stimulated emission of radiation (LASER), which have a biomodulating effect on cellular functions and can promote analgesic effects, the modulation of inflammation and edema, and tissue repair more recently, studies have shown favorable outcomes in terms of muscle performance [12].

Additionally, LLLT is a form of biostimulation that includes increased blood circulation, vasodilatation, accelerated healing of injured tissues. Its low cost and more durable equipment, making it a practicable treatment option, especially for children [13, 14].

An infrared laser with a wavelength of 980 nm has an abundant affinity for cellular mitochondria, leading to increased oxygen consumption, potential of the mitochondrial membrane, and ATP synthesis [15].

Another modality of treatment is physical therapy, which has a major role through diverse approaches, such as electrical stimulation, stretching, massage, facial exercise, relaxation techniques and biofeedback. These approaches are effective, inexpensive, safe and have long-lasting effects on bruxism, particularly in children [16].

As a means of unwinding, Jacobson created the gradual muscular relaxation technique. The progressive muscle relaxation method has been shown to be highly effective in the literature as a supportive therapy for the treatment of hyperactive muscles and bruxism in children [17, 18].

According to our knowledge no study has been conducted in the literature comparing the effect of behavioral management, physical therapy and use of LLLT for management of various adverse outcomes of bruxism in children.

Specific objectives of this study is to compare and evaluate changes in pain, mouth opening and muscle activity in children with bruxism after using laser acupuncture versus the relaxation technique as a physical therapy intervention and sleep hygiene as a control therapy.

Hypotheses of this research are whether the management of bruxism in children has value for its progress and which modality (laser acupuncture, physical therapy or physical therapy) is best for its management based on their promising results.

## Methods

### Study design

**Fig. 1:**
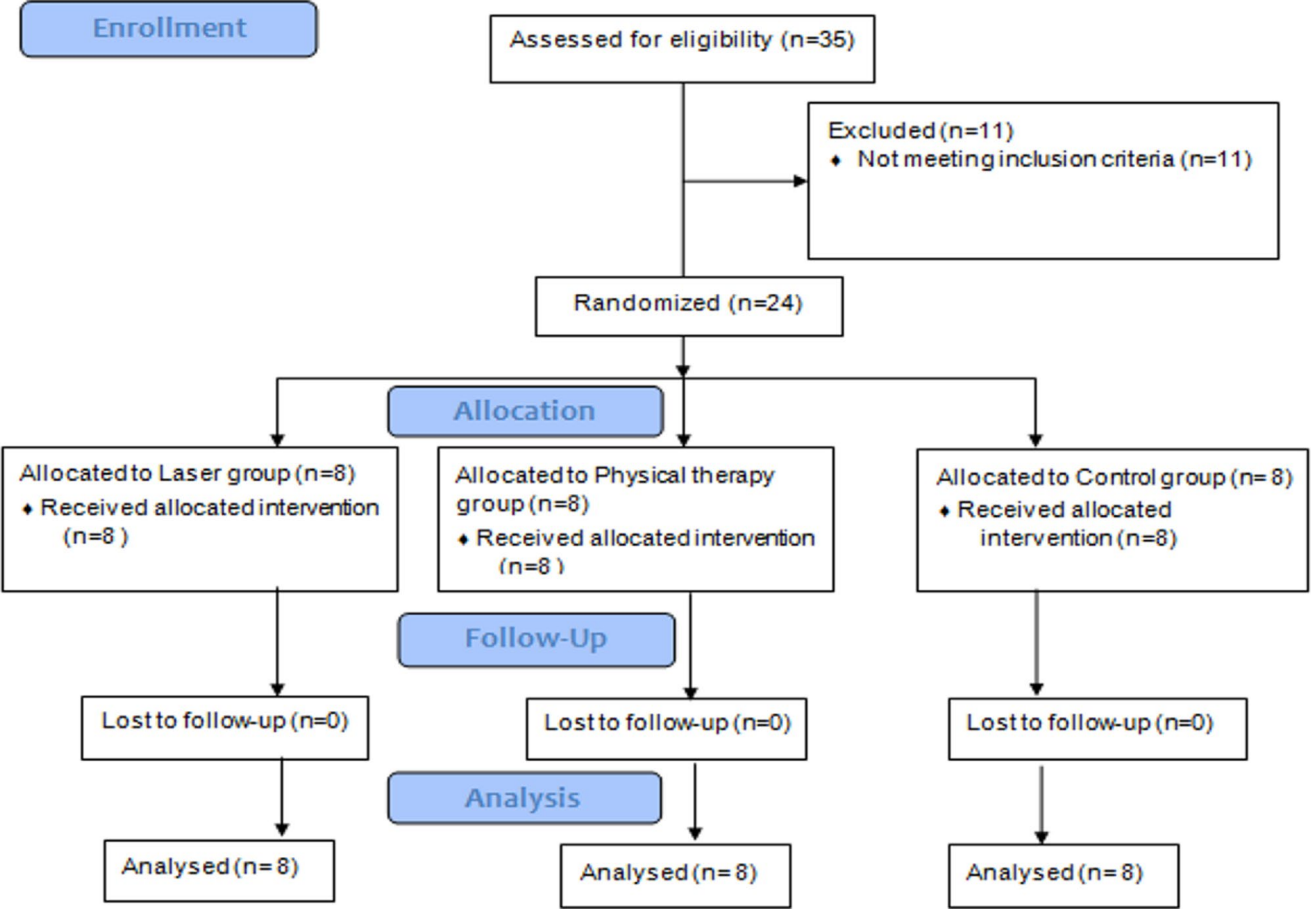
Showed participants flow diagram

This study was designed as a single-blinded randomized controlled trial (RCT) in compliance with the Consolidated Standards of Reporting Trials (CONSORT) statement of 2010 for planning and reporting clinical trials [19]. Thirty-five participants were enrolled in the study. Twenty-four patients were randomly assigned, received the intended treatment, and were subsequently analyzed for different outcomes during the different stages of the study; as shown in the CONSORT 2010 flowchart presented in Fig. 1.

### Ethical approval and registration

This in vivo study was approved by the Medical Research Ethical Committee at the National Research Centre, Egypt, with approval number 03440423 on April 6, 2023, in accordance with the ethical standards laid down in the 1964 declaration of Helsinki and its later amendments [20]. The study was registered on ClinicalTrials.gov on November 12, 2023, with ID: NCT06131879.

### Sample size calculation

In a recent study [21], the mean visual analog scale (VAS) score at 6 months was 2.5 ± 2, whereas the value was 0.3 ± 0.2, and the G power statistical power analysis program (version 3.1.9.4) was used for sample size determination [22]. A total sample size of *n* = 16 for the intervention groups (subdivided into 8 in each group) was sufficient to detect a large effect size (d) = 1.54 at 6 weeks, with an actual power (1-β error) of 0.8 (80%) and a significance level (α error) of 0.05 (5%) for the two-sided hypothesis test. Similarly, 8 children were included in the control group. We have chosen this particular effect size as it is almost the same period of follow up of primary outcome (VAS at 6 weeks).

### Setting and location

Children were recruited from outpatient dental clinics at the National Research Centre, Egypt. Children were recruited for diagnosis between June 2023 and January 2024. The follow -up period ended in March 2024.

### Participants

Thirty-five children aged between six and twelve years were referred to the Pedodontic Department during the study period. The principal investigator examined the children for eligibility for this study; eleven children were excluded because they had some neurological disease, such as epilepsy. Twenty-four children were considered eligible for our study according to the following criteria:

### Inclusion criteria

Six to twelve-year-old children with no or few caries, whose teeth are in normal occlusion with clinical dental wear, were included in our study. Clenching or grinding was reported by the parents by asking them if they noticed any grinding or clicking sounds coming from their child’s mouth during sleep at night. The children enrolled in the study were diagnosed with bruxism according to the criteria of the American Association of Sleep Medicine [23].

### Exclusion criteria

All children with any physical, neurological or psychological disease or any children who received any previous treatment or medication for bruxism, or who were suffering from TMJ or gastrointestinal disorders were excluded from the study.

### Informed consent

Before starting the intervention, written informed consent was obtained from the parents after brief explanations of the procedures were provided in a simple language. A verbal assent was also obtained from the child preoperatively.

### Randomization

The children were assigned to the control group (this group received behavioral strategies and sleep hygiene instructions with no treatment applied), Study group 1 (this group received LLLT acupuncture via a diode laser) or Study group 2 (this group received physical therapy (relaxation therapy). With a 1:1 allocation ratio, a random sequence was created via the website www.random.org, which was accessed on 18 April 2023. Thus, the children were assigned to 3 groups: Group 1; the LLLT acupuncture group with a diode laser = 8, Group 2; the physical therapy group (relaxation therapy) = 8 and Group 3the control group with no treatment applied (behavioral strategies and sleep hygiene only were performed) = 8.

### Allocation concealment

MF generated the random sequence in on the website (random.org). The allocation sequence was kept in opaque sealed envelopes with MF so the allocation was concealed. Operator MA, MM selected the patients and NE released the allocation for each patient at the treatment appointment. MA, MM provided patient treatment.

### Blinding

In the present study, a single-blinded approach involving examiners, assessors and statisticians was implemented. However, the treating clinician and children could not be blinded because of the difference in the interventional nature between laser therapy and physical therapy. Moreover, evaluation of treatment outcomes was carried out by a well-trained researcher who was unaware of the intervention performed in each case.

#### Clinical procedures

### Grouping of samples

Group 1: LLLT acupuncture group treated with a diode laser.

Group 2: Physical therapy was performed (relaxation therapy).

Group 3: Control group with no treatment applied (behavioral strategies and sleep hygiene only were performed).

### Diagnosis of bruxism in subjects

The clinical diagnosis of bruxism is a multifaceted procedure that involves various examinations, including subjective observations and analyses of medical history, clinical examinations, and assessments and recordings of muscle activity via surface electromyography (SEMG) [24].

The diagnosis is typically based on family members, who define the distinctive sounds produced by tooth grinding at sleeping time [25].

#### Clinical symptoms: (Noninstrumental diagnosis)

##### Child history

Guardian, or sibling hearing regular audible teeth-grinding from the child while sleeping for at least 3–5 nights per week in the last 3–6 months.

##### Clinical assessment

*Nonphysiologic* wear of teeth, hypertrophy and tenderness of the temporalis and masseter muscles, and the presence of pain or discomfort related to the temporomandibular joint (TMJ) (temporary, related to awaking from sleep or causing headache).

Data such as sugar content and screen time per day were recorded at baseline. We considered consuming 1 chocolate bar (our unit of measurement in our study asked to the parents) or its equivalent from sweets as once sugar consumption.

At baseline and after 2 months of intervention, maximum mouth opening was recorded via a digital caliber; the degree of TMJ pain was recorded via a visual analog scale (VAS) via simple images categorized the pain as no, mild, moderate or severe. VAS Pain Score Categories: [26, 27].

1. No Pain:

- Score Range: 0.
- Description: The patient experiences no pain at all.

2. Mild Pain:

- Score Range: 1–3.
- Description: Pain is present but not interfering significantly with daily activities or comfort.

3. Moderate Pain:

- Score Range: 4–6.
- Description: Pain is more noticeable, can interfere with concentration and some activities, but the patient may still be able to function.

4. Severe Pain:

- Score Range: 7–10.
- Description: Pain is intense, limiting, or disabling, often requiring immediate intervention or treatment to manage.
- Tooth grinding or clenching while sleeping;
- One or more of the following:

#### The surface electromyography device: (Instrumental diagnosis)

The evaluation was performed in the EMG laboratory of the faculty of physical therapy, Misr University for Science and Technology, Egypt.

### Measurement instruments

The patients were assessed before and after treatment via a surface electromyography device (Model: NS062201.001; Manufacturing Company: NEURO SOFT; Country: Russia). The maximum bite of the patients was recorded from the masseter and temporalis muscles (right and left).

The assessment was achieved by the device hardware (screen, CPU speed and RAM memory size, hard drive, loud speakers, printer), and software (analysis software, recorder, database support, operating system) was connected to a computer via a USB cable. This cable allows the user to upload data and power the device.

*Set up*: (according to the European concerted Surface EMG Non-Invasive Assessment of Muscles (SENIAM protocol [28]. Setting up the patients: After a brief explanation of the assessment and the device for the children, the child was instructed to keep the posture erect on a fixed wooden chair to avoid any movement artifacts, and then the electrode was placed on cleaned skin with alcohol swap (3*6 cm) with percentage 70%. The conductive media (gel) was placed on the electrode before placement and the reference electrode was attached first to the nose (silent area) while the recorded electrode was placed on the selected muscle away from the neighboring muscles to avoid signal overlap. The position of the electrodes was over the muscle belly encompass the muscle endplate along the longitudinal midline of desired muscle parallel to muscle fiber. Inter-electrode Distance was 2 cm (20 mm) between active muscle and reference electrode. The patient file was created including the name, patient number, sex, diagnosis and therapist name. The targeted muscles were selected (masseter and temporalis; right and left. We get SEMG signal during rest and maximum biting (clenching) and get recordable waveform of all parameters (minimum, maximum, mean and normal amplitude and polyphasicity) scored with machine quantitatively and automatically analyzed into table so to assess muscle force during rest and maximum biting (clenching). For comparison between the three interventions we took the maximum amplitude (MVC). Recording duration was 1 min and surface contact area of each active electrode was 1*1 cm small contact area to less cross talk other muscle activity. MVC readings were taken at baseline and after 2 months of intervention for all groups. SEMG assessment was performed by a professional electro diagnosis consultant with 25 years of experience in dealing with EMG devices [29].

### Treatment modalities

#### Group 1: Laser group

##### Laser application protocol

Photobiomodulation was performed as twelve laser sessions were performed with a frequency of two sessions per week with the diode laser device; (Doctor Smile, Wiser, LAMBDA SpA, Italy) using the flat top handpiece model AB 2799.

The laser parameters: Wavelength were as follows: wavelength: 980 nm, power: 0.3 W handpiece: flat top with aperture diameter: 1 cm and energy density: 23 J/cm^2^ 60 s per point. The child was positioned with the Frankfurt plane parallel to the floor in a noiseless place without any disturbing sounds. The delivery handpiece of the laser was wrapped with a plastic cover to avoid cross-infections and for hygienic reasons.

Three acupuncture points on the masseter muscle (upper, middle, and lower parts) and 3 points on the temporalis muscle (anterior, middle and posterior parts) on each side of the face were irradiated with a laser [30]. The child was instructed to stay stationary, and the handpiece was held by the operator perpendicularly and in noncontact mode with a distance of 1 mm from the point to be irradiated for 60 s.

#### Group 2: Physical therapy group

- The patient was given an explanation of the processes and aims of treatment prior to beginning Jacobson progressive muscle relaxation, tailored to their level of knowledge.
- The patients received a brief description of how the device would be operated on and how the process would not be painful.
- During training, the patient was instructed to maintain a comfortable position on the plinth, with their head passively retracted into an erect position and their sternum elevated.
-A total-12 sessions (2 sessions per week) of progressive muscle relaxation techniques were performed for each child.
- The child followed 5 stages from Jacobson Progressive Muscle Relaxation for both the masseter and temporalis muscles bilaterally Table 1 [31, 32].

**Table 1:** Jacobson progressive muscle relaxation technique stages

Region	Action	Tensing time	Relaxation time
Facial muscles	Elevate the eyebrow. Close eye firmly Clench teeth Then hold the Tension	5 s	10 s
Hands	Clench fists firmly	5 s	10 s
Arm	Bend each arm to the elbow and tense the biceps.	5 s	10 s
Neck and shoulder	Push the head backward against cushion	5 s	10 s
After exercise	Relax whole body Completely with deep breathing		2 min

#### Group 3: Control group

No treatment was applied; behavioral strategies were performed only for both children and their parents to break the SB habit. Behavioral strategies include relaxation and improved sleep hygiene.

Relaxation before sleep was advised to avoid watching screens on either mobile phones or television.

Sleep hygiene measures included keeping good aerated and quiet bedrooms. Sleep instructions were given to the child and his parents, which included relaxation and avoidance of stress for the child before sleep and reading a story and listening to calm music before sleep. Avoiding chewing gum, pen or any other object with drinking water and milk cup before night tooth-brushing by placing a warm towel on the child’s face right or left would be beneficial. Muscle massage should be performed to enhance child relaxation.

The objective of these measures is to minimize the effect of psychological stress on sleep bruxism [7, 33].

**Fig. 2:**
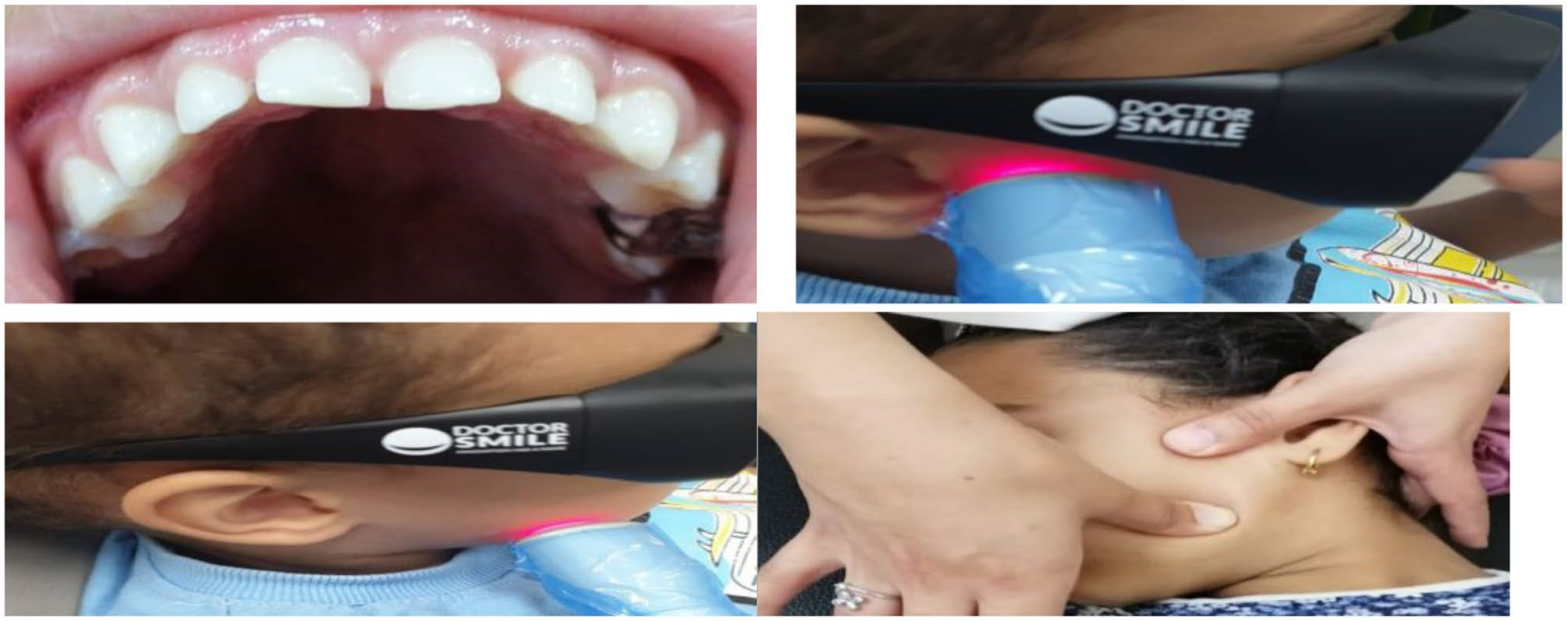
Shows examples of study cases (**a**) Attrition of teeth before study (**b**) Laser acupuncture of temporalis muscle (**c**) Laser acupuncture of masseter muscle (**d**) Physical therapy of masseter muscle

Examples of cases are shown in Fig. 2.

### Outcome assessment

Patients in all the groups were assessed at baseline and after 2 months of intervention for the degree of TMJ pain (primary outcome); (VAS) Simple images were used to categorize the pain into no, mild, moderate or severe, and maximum mouth opening was recorded via a digital caliber and muscle activity in terms of MVC via an SEMG device as secondary outcomes.

### Statistical analysis

Data management and statistical analysis were performed via the Statistical Package for Social Sciences (SPSS) version 20. Numerical data were summarized using means, standard deviations, confidence intervals, medians and ranges. Data were explored for normality by checking the data distribution and using Kolmogorov‒Smirnov and Shapiro‒Wilk tests.

On the basis of the normal distribution of maximum mouth opening data, groups were compared via one-way ANOVA, followed by Bonferroni post hoc correction for pairwise comparisons. A paired t test was used for intra (within) group comparisons.

The Kruskal‒-Wallis test, followed by the Mann‒Whitney test as a post hoc test was used to compare MVCs between groups on the basis of the nonparametric distribution of these data, whereas the Wilcoxon signed rank test was used to compare pre and post values within the same group.

Qualitative data (gender and VAS score) are expressed as counts and percentages.

The amount of difference between the pre- and postoperative values was calculated as follows: Difference = value after-value before. All p-values are two-sided. P-values ≤ 0.05 were considered significant.

## Results

The participants in the laser group consisted of 6 females and 2 males (25%); compared with 2 females (25%) and 6 males (75%) in in the physical therapy group, an equal sex distribution (50%) was observed in the control group (4 females and 4 males). The difference between groups was not statistically significant (*p* = 0.135).

### The demographic data and clinical characteristics of each group are shown in Table 2

**Table 2:** Descriptive statistics and comparison between groups regarding age, screen time (hour/day) and sugar time (sweet/day)

		Mean	Std. Dev	Median	95% Confidence Interval for Mean		Min	Max	P value	Effect size (Partial Eta Squared)	Power
					Lower Bound	Upper Bound					
Age^a^	Laser	7.25	2.55	7.50	5.12	9.38	4.00	10.00	0.541 ns	0.057	0.142
	Physical therapy	7.75	2.87	7.00	5.35	10.15	5.00	12.00			
	Control	6.50	0.53	6.50	6.05	6.95	6.00	7.00			
Sugar time^K^	Laser	1.50	0.93	2.00	0.73	2.27	0.00	2.00	0.829 ns	0.000	0.050
	Physical therapy	1.50	0.93	1.00	0.73	2.27	1.00	3.00			
	Control	1.50	0.53	1.50	1.05	1.95	1.00	2.00			
Screen^K^ Time	Laser	3.00	1.69	3.00	1.59	4.41	1.00	5.00	0.511 ns	0.143	0.325
	Physical therapy	7.00	6.46	6.50	1.60	12.40	1.00	14.00			
	Control	7.00	5.35	7.00	2.53	11.47	2.00	12.00			

Significance level *p* ≤ 0.05, ns = non-significant, a = ANOVA test, K = Kruskal Wallis test

### Pain (visual analog scale; VAS)

In the laser group, moderate pain was recorded preoperatively in all 8 patients, whereas 100% mild pain was recorded postoperatively. This difference was statistically significant (*p* = 0.000).

The physical therapy group, moderate pain was recorded preoperatively in all 8 patients, whereas 100% mild pain was recorded postoperatively. This difference was statistically significant (*p* = 0.000).

Control group: Before and after treatment, all 8 patients (100%) experienced moderate pain, with no significant difference throughout the study (*p* = 1).

### Maximum mouth opening

**Pre-operatively**, there was no significant difference between groups (*p* = 0.062).

**Post-operatively**, the highest mean value was recorded in physical therapy group (38 ± 2.62), followed by laser group (36.8 ± 1.8), and with the lowest value recorded in control group (34.5 ± 2.67). ANOVA test revealed a significant difference between groups, with the value recorded in physical therapy group being significantly higher than control group (*p* = 0.025). Post hoc test revealed that laser group was not significantly different from each of the other groups.

#### Amount of difference from pre to post-operative

**Fig. 3:**
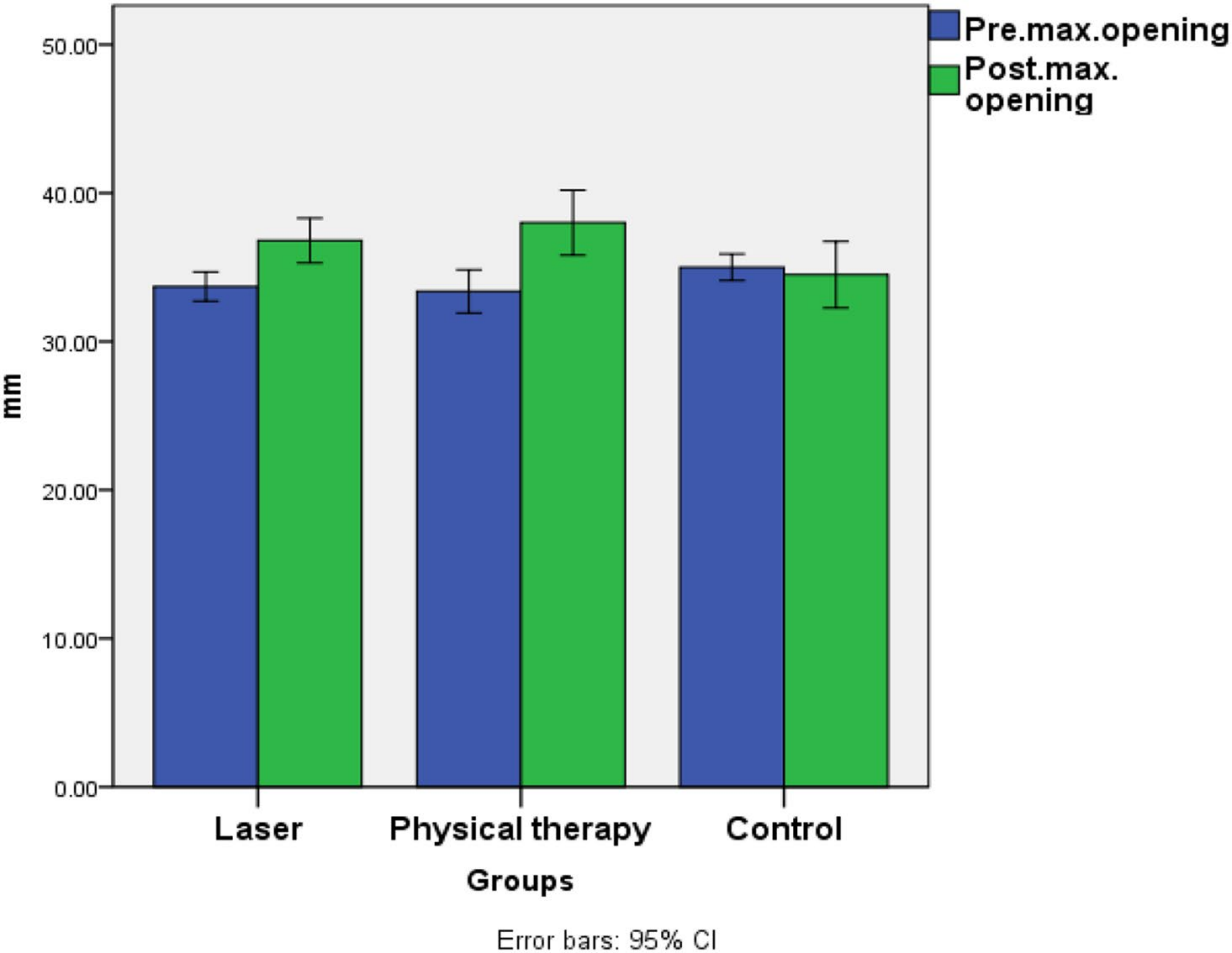
Bar chart illustrating mean value of maximum mouth opening (mm) in different groups

The highest mean value was recorded in the physical therapy group (4.63 ± 2.31), followed by the laser group (3.1 ± 0.72), with the lowest value recorded in the control group (1 ± 0). ANOVA revealed a significant difference between the groups, with the values recorded in the physical therapy and laser groups being significantly greater than those recorded in the control group (*p* = 0.005). A post hoc test revealed that the laser group was not significantly different from the physical therapy group; Fig. 3.

### Muscle activity (overall MVC)

The results are summarized in Table 3. Preoperatively, there was no significant difference between the groups (*p* = 0.591). Postoperatively, the control group had a significantly (*p* = 0.000) greater median value (1662.13), than did the physical therapy group (651.75) and laser group (526.63). Post hoc tests revealed no significant difference between the laser and physical therapy groups. The control group presented a median value of increase (1003.88), whereas the laser group and physical therapy group presented median values of decrease (-250.75 and − 248.13, respectively). Post hoc tests revealed no significant difference between the laser and physical therapy groups.

**Table 3:** Descriptive statistics and comparisons between groups regarding overall (MVC) (Kruskal-Wallis test)

MVC overall		Mean	Std. Dev	Median	95% Confidence Interval for Mean		Min	Max	Test value	P value	Effect size (Partial Eta Squared)	Power
					Lower Bound	Upper Bound						
Pre	Laser	890.56	396.27	816.88	559.27	1221.85	497.25	1431.25	1.05	0.591 ns	0.150	0.342
	Physical therapy	980.06	440.26	929.00	612.00	1348.13	494.00	1568.25				
	Control	658.25	77.51	658.25	593.45	723.05	585.75	730.75				
Post	Laser	636.63	300.84	526.63 ^b^	385.12	888.13	392.75	1100.50	15.55	0.000*	0.645	1.000
	Physical therapy	629.19	202.61	651.75 ^b^	459.80	798.57	374.00	839.25				
	Control	1662.13	558.71	1662.13 ^a^	1195.03	2129.22	1139.50	2184.75				
Difference	Laser	-253.94	130.31	-250.75 ^y^	-362.88	-145.00	-409.75	-104.50	15.63	0.000*	0.800	1.000
	Physical therapy	-350.88	278.19	-248.13 ^y^	-583.45	-118.30	-787.25	-120.00				
	Control	1003.88	481.20	1003.88 ^x^	601.58	1406.17	553.75	1,454.00				

Significance level *p* ≤ 0.05, *significant, ns = nonsignificant

Post hoc test: within the same comparison, medians sharing the same superscript letter are not significantly different

No harms or intended effects were recorded in any group such as any laser accidents such as eye injury due to accidental exposure to laser in group one, or muscle fatigue or pain that may occur after physical therapy in some rare cases in group two.

## Discussion

Bruxism is defined as a dental habit pertaining to tooth contact caused by frequent, nonnutritive, inadvertent and unaware muscular activities of the masticatory system [34]. Bruxism is the most prevalent oral habit in children worldwide [35], with mean prevalence 19.5% [36, 37]. While in Egypt it was found to be from 17.6 to 19.1% [38, 39].

Controversy concerning early intervention in juvenile bruxism exists among experts. Some professionals assume that three to five years is the time of occlusal wear to aid in alveolar growth and development, and from nine to ten years, the presence of bruxism decreases with age and does not proceed to the adult type [30, 40].

However, sleep problems such as bruxism are more common in childhood than before; and may have serious adverse effects. These adverse effects include breathing problems, snoring, restless and noisy sleep dental defects such as wearing facets and myofacial pain, limited mouth opening, masticatory muscle hypertrophy and/or tenderness,; thus, pediatric dentists must monitor this bad habit and provide effective tools for diagnosis and management [41, 42].

Surface Electromyography (SEMG) is commonly used to evaluate masticatory activity in children during rest, function and monitoring intervention effects [43]. SEMG readings, is valid and comparable to standard polysomnography (PSG) recordings, providing strong evidence of sleep bruxism; [44, 45]. The adoption of PSG for detecting juvenile bruxism has several drawbacks, such as its high cost and inability to cooperate [46, 47].

No consensus has been reached regarding which treatment option is effective for the management of juvenile bruxism compared with other options which includes pharmacotherapy, stabilizing splints, psychological treatment, physiotherapy and low-level laser acupuncture [48–50].

An occlusal splint is a widely used option for the management of bruxism in adults and may be considered the gold standard [51]. Nevertheless, there are impediments for its application in children including the need for child and parent compliance and may affect alveolar growth and eruption of permanent teeth [47, 49]. Additionally, rapid maxillary expansion was found to reduce SB activity in children, but this option is indicated for those who suffer from maxillary deficiency [52].

Medications such as hydroxyzine and diazepam have been used for the management of bruxism in children but results were not significant in improving bruxism signs and symptoms with various adverse effects [53, 54].Also Melissa officinalis L revealed no significant effect in managing bruxism in children [55].

The increased sugar consumption and screen time in most children in this study are in accordance with a study that concluded that as their consumption increased, the rate of bruxism in children increased [56].

Standard for screen-time according to the guidelines of WHO, is less than 2 h/day) [57]. As average-sized bar of milk chocolate (around 45 g) contains about 25 g of sugar. While sugar time standard according to the guidelines of the American Academy of Pediatrics is less than 25 g (about 6 teaspoons) of added sugar per day for children 2 years of age and older. So we considered consuming 1 chocolate bar (our unit of measurement in our study asked to the parents) or its equivalent from sweets as once sugar consumption. While consuming more than this quantity is considered as (increased sugar consumption) [58].

Bruxism can cause masticatory muscular pain or temporomandibular joint pain. Previous studies have shown that subjects with severe bruxism have greater TMJ pain than do those with moderate bruxism [59, 60]. Assessment of pain severity using different scales is relatively prejudiced. However, the visual analog scale (VAS) is a commonly used method of measuring TMJ pain severity at baseline and at follow-up visits [61].

Compared with the experimental groups, the results of the control group in this study revealed no change in the pain threshold, an increase in EMG readings and a decrease in maximum mouth opening. The results highlight the need to manage juvenile bruxism and not leave it to be corrected on its own by being older.

These results contrast with those of a previous study [62], which revealed no difference between the control and experimental groups. These differences may be attributed to different age groups. Photobiomodulation therapy has been suggested for temporomandibular disorders, including bruxism in children [47].

Acupuncture has been widely chosen to control bruxism, causing a reduction in the activity of the temporalis and masseter muscles. Stimulation of specific acupuncture points via infrared light or a low-level laser may affect circulation kinetics and enhance the reduction in the activity of hyperactive muscles resulting in TMJ pain. In children, laser acupuncture is the preferred method because it is safe, noninvasive, and free from pain in a relatively fast treatment sessions [63]. Additionally, photobiomodulation over acupuncture points was adopted in subjects affected by Down syndrome, and relieved the symptoms of bruxism in these children [64, 65].

In this study, the improvement in pain and muscle activity from the preoperative values to the postoperative values was statistically significant. These results could be attributed to its novel and safe biostimulatory effect on acupuncture points of the temporalis and masseter muscles for the relief of various effects [66].

These results are in agreement with those of a randomized controlled trial [65] that revealed a significant difference between the number of children experiencing pain and increased muscle activity related to laser therapy over acupuncture points before and after intervention.

Physiotherapy modalities relying on medical and psychological models can be directed toward managing the adverse effects of bruxism as a conservative option. Physical therapy was found to be an effective treatment option for bruxers with myofascial pain [67]. The combination of exercise and psychotherapy, which is based on cognitive behavior therapy principles within physical therapist practice, is strongly adopted [21, 68].

The children in the physical therapy group in this study showed a reduction in VAS and EMG readings of the MVC for both selected muscles and an increase in maximum mouth opening. A statistically significant difference from the preoperative value to the postoperative value was recorded. These favorable results could be attributed to enhanced response of children to massage and relaxation techniques, which are suitable for their age.

These results are in agreement with the work of a previous study [69] that reported a reduction in pain in the massage group. Additionally, a significant reduction in the VAS score was observed in the physical therapy group [70].

Similarly, in another study, significant improvements were observed in muscle pain and maximum mouth opening, in addition to the superior effect of combining physical therapy interventions (massage, relaxation and exercise) [21, 60].

Another study [71] reported a reduction in the incidence of sleep bruxism but the difference was not significant. The difference in the results may be attributed to the different age groups of the participants (3–8 years).

The first limitation is the lack of an occlusal splint as the gold standard group. The reason for this was the inability of young children to wear such bulky appliances. The second limitation was that relaxation is the sole physical therapy technique, as other techniques, such as biofeedback require older patients to cooperate. PSG was not used because it was unavailable and clinically cannot be used with children.

Small sample size, short follow-up time, and uncertainty of long-term efficacy of the intervention are significant limitations of our study.

On the basis of the results of this study, we recommend early detection of signs of bruxism in children during routine dental examination. In addition to limiting screen and sugar intake during the daytime, sleep bruxism has adverse effects in addition to encouraging sleep hygiene and muscle relaxation before sleep, together with coordination among pediatric dentists, physiotherapists and laser specialists in cases of juvenile bruxism. In the future we recommend making multiple studies on bruxism in children with larger sample size and extended follow up period using psycho-social parameters.

## Conclusions

Therefore, we can conclude that laser acupunture and physical therapy are promising options for treating SB in children in terms of pain, mouth opening and muscle activity in comparison to sleep hygiene. Enhanced effects could be assured by applying the intervention to larger sample size and longer follow up period.

## Data Availability

The datasets used and/or analyzed during the current study are available from the corresponding author upon reasonable request. All efforts were made to avoid compromising an individual’s privacy.

## Abbreviations

TMJ: Tempro-Mandibular Joint
VAS: Visual Analogue Scale
SENIAM: Surface EMG Non-Invasive Assessment of Muscles
MVC: Maximum Voluntary Contraction
TMDs: Tempro_Mandibular Disorders
SB: Sleep bruxism
OST: Occlusal Splint Therapy
LLLT: Low-Level Laser Therapy
PBM: Photobiomodulation
LED: Light Emitting Diode
LASER: Light Amplification by Stimulated Emission of Radiation
RCT: Randomized Controlled Trial
CONSORT: Consolidated Standards of Reporting Trials
SEMG: Surface Electromyography
SPSS: Statistical Package for Social Sciences
PSG: Polysomnography
